# Design and Fabrication of High-Frequency Resonant Micro-Accelerometer Based on Piezoelectric Stiffening Effect

**DOI:** 10.3390/mi17040483

**Published:** 2026-04-16

**Authors:** Ankesh Todi, Hakhamanesh Mansoorzare, Reza Abdolvand

**Affiliations:** Department of Electrical Engineering, University of Central Florida, Orlando, FL 32817, USA; hakha@ucf.edu

**Keywords:** MEMS resonant accelerometer, TPoS, high frequency, fabrication, suspended structures

## Abstract

**Highlights:**

**What are the main findings?**

• Introducing piezoelectric stiffening as a mechanism to decouple operation frequency of resonant accelerometers from the mass-spring acceleration transduction mechanism.
• Feasibility of fabricating planar metallic capacitive read-out using photoresist as sacrificial layer.

**What are the implications of the main findings?**

• Enabling battery-less resonant vibration sensors.
• Reducing barrier to entry for low-lost diagnostic sensor nodes.Enabling battery-less resonant vibration sensors.

**Abstract:**

In this work, a novel approach for implementing a resonant micro-accelerometer is demonstrated that may extend the operating frequency of such devices to several tens of MHz, which may enable direct wireless signal transfer. The proposed resonant accelerometer consists of a hybrid structure: a piezoelectric micro-resonator and a capacitive mass-spring (CMS) system (that are mechanically separated but electrically interconnected). The sensor utilizes the piezoelectric stiffening mechanism, which translates the acceleration-induced displacement of the capacitive mass-spring (CMS) structure into a shift in the resonance frequency of the interconnected resonator. The operating principle is elaborated upon in detail, supported by simulation and experimental results. Additionally, a novel fabrication technique is presented to realize a suspended fixed bi-layer electrode for the CMS in which a hardened layer of photoresist is utilized as a sacrificial layer. The experimental sensitivity of a fully functional device is reported to be ~6 Hz/g at 25 MHz (~0.23 ppm/g), which closely matches the simulated sensitivity of ~7 Hz/g (~0.278 ppm/g) for the fabricated capacitive gap of ~7 µm.

## 1. Introduction

Microelectromechanical system (MEMS)-based accelerometers have become ubiquitous across diverse applications, including consumer electronics, space and defense systems, and automotive technologies [1,2,3]. Their compact size, compatibility with integrated circuit microfabrication, high resolution, and scalability for mass production render them highly desirable for such numerous applications [4,5]. MEMS accelerometers utilize various transduction techniques, including capacitive [6], piezoresistive [7], and optical [8], to produce either a DC electrical signal or a frequency shift, as in resonant accelerometers as an output. Although alternative technologies like fiber-optic vibration sensors deliver exceptional resolution, their form factor may limit their practicality in applications where compact solutions are required [9,10].

Amongst other MEMS accelerometer technologies, resonant accelerometers have garnered considerable interest for their quasi-digital output, high sensitivity, and superior scale factor [11,12,13,14]. Measuring acceleration through shifts in resonance frequency offers a high signal-to-noise ratio and dynamic range while facilitating simplified readout circuitry and potentially reduced power consumption. Resonant accelerometers typically integrate a resonator and a proof mass on the same mechanical platform, where the stiffness of the resonant structure is altered by the acceleration-induced displacement of the proof mass. Therefore, the sensitivity of the sensor is dependent on the ratio of the proof mass to the stiffness of the resonant structure. However, this intrinsic dependence ultimately limits the targeted resonator stiffness and consequently the resonance frequency of the sensor, thus limiting this critical performance metric [15,16,17,18,19,20].

Recent progress in resonant accelerometer designs has focused on improving performance through either novelty in the design of the force-loading structures or the resonator designs [21]. The force-loading structure typically includes a proof mass that, under acceleration, changes the resonator’s stiffness and, consequently, its resonance frequency. Typically, levers are implemented to amplify the force exerted to the resonant body in response to the displacement of the proof mass.

Several different resonator designs have been studied to enhance performance parameters such as sensitivity, scale factor, and resonance frequency. One widely used design is the bridge resonator, which typically features a clamped–clamped beam [22] or a fishbone-shaped resonator [23,24,25]. Another prominent design is the double-ended tuning fork (DETF) [26,27]. Despite these efforts, a persistent feature in MEMS accelerometer technology is the limited operational frequency (kHz range). This limitation arises because to improve the acceleration sensitivity with a given size of proof-mass, the stiffness of the resonant structure must be reduced, thereby constraining its resonance frequency range.

High-frequency operation (in the MHz regime) is desirable since it could potentially offer advantages, such as a higher signal-to-noise ratio, and the potential for wireless data transmission without requiring complex electronics or large antennas. Recent bulk acoustic wave (BAW) accelerometers have demonstrated promising outcomes in partially addressing the low operational frequency limitation by inducing an acceleration-sensitive frequency shift in a piezoelectrically actuated resonator through an extremely small capacitive gap between the moving resonant body and the fixed surrounding frame [28]. Even though such accelerometers can operate at higher frequencies (few MHz), the frequency scaling is limited by the efficiency of the underlying electrostatic frequency tuning mechanism at high frequencies for reasonably sized capacitive gaps.

Despite ongoing efforts, high-frequency MEMS accelerometers (operation at 10 s of MHz) remain very challenging to implement. Bridging this gap shifts the signal away from common low-frequency noise sources, significantly improving the signal-to-noise ratio and overall measurement fidelity. This approach not only supports the miniaturization of the sensor but also lays the foundation for a new class of high-performance accelerometers suitable for applications demanding a compact size, high precision, and seamless wireless connectivity.

Recently, our group introduced a novel design paradigm that enables high-frequency resonant micro-accelerometers [29]. Through a combination of theoretical modeling, device simulation, and experimental validation, we expand on our earlier work and introduce a novel strategy to overcoming the conventional trade-off between frequency and sensitivity. Particularly, we offer an improved fabrication process that promises reliable delivery of a high-frequency resonant accelerometer that operates in the 10s of MHz regime. The following sections outline the sensor’s design principle, simulation, fabrication process—with an emphasis on large, suspended structures—and experimental results.

Finally, we conclude by discussing the implications of these findings for the future of MEMS accelerometer technology.

## 2. Theory of Operation

**Figure 1:**
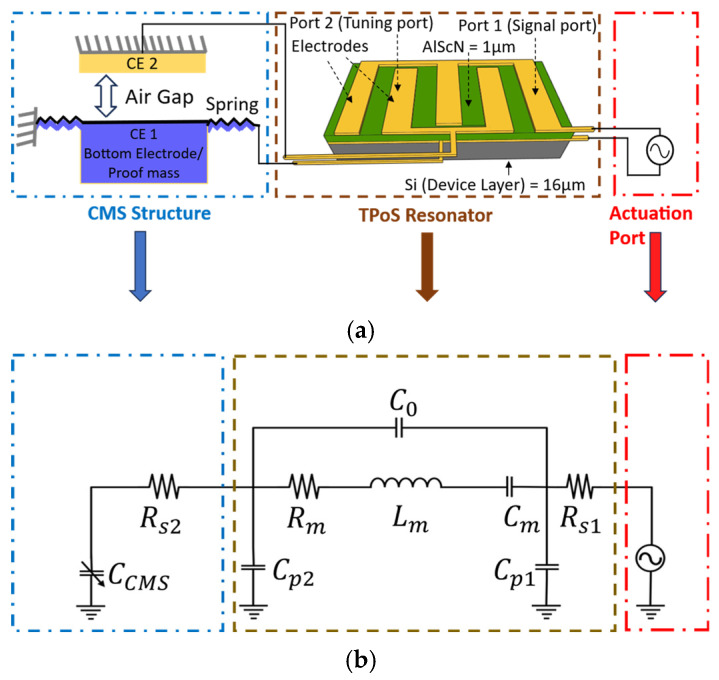
(**a**) The schematic representation of the resonant accelerometer concept presented in this work. A 2-port thin-film piezoelectric-on-silicon resonator is connected to a capacitive mass-spring (CMS) structure from one port, inducing an acceleration-induced frequency shift in the resonator measured from the second port. (**b**) An equivalent modified Butterworth–Van Dyke (mBVD) model of the 2-port resonator captures the acceleration-induced frequency modulation in the electrical domain.

The effective elastic modulus ($E_{Eff}$) of a piezoelectric material can be dynamically modified by the electrical boundary conditions at its surfaces. The proposed accelerometer of this work utilizes this property (i.e., the piezoelectric stiffening effect) to induce a shift in the resonance frequency of a thin-film piezoelectric-on-silicon resonator [30,31,32,33,34] by connecting an acceleration-activated variable capacitance (i.e., the capacitive mass spring system) across a set of electrodes on the resonator (Figure 1).

**Figure 2:**
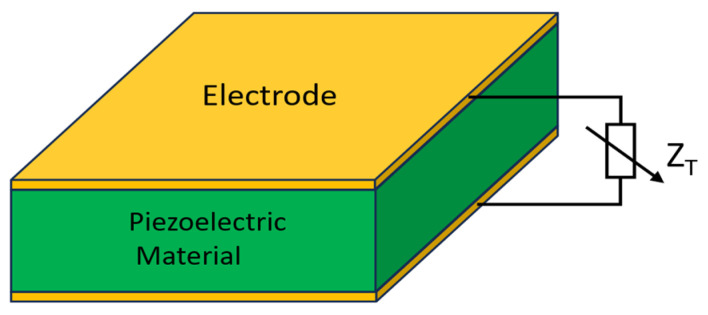
Variable shunt impedance is electrically connected to the electrodes covering the piezoelectric material.

The effective elastic modulus of a block made of piezoelectric material and terminated with metallic electrodes can be tuned by the impedance connected to the electrodes (Figure 2), as described in (1) [35,36]:

(1) $${E_{Eff}=\left(S^{E}-j\omega \frac{d^{2}A}{t}\left(Z_{T}∥Z_{P}\right)\right)}^{-1}$$

where $E_{Eff}\;$ is the effective modulus, $S^{E}\;$ is the material compliance, *d* is the piezoelectric coefficient, A is the area of the electrode, t is the thickness of the piezoelectric material, $Z_{T}$ is the shunt impedance and $Z_{P}$ is the intrinsic capacitive impedance of the piezoelectric slab.

From (1), it can be inferred that the effective stiffness is minimal at short-circuit termination and conversely, the effective modulus reaches a maximum at open-circuit termination.

The equivalent modified Butterworth–Van Dyke (mBVD) model of the proposed sensor shown in Figure 1b captures the described operation principle, where one resonator port (Port 1) is reserved for actuation and monitoring of the resonance frequency, and the other port (Port 2) is connected to the variable capacitor causing the frequency shift.

The generalized “d” term represents the adaptivity of the hybrid accelerometer to incorporate any two-port resonator, such as bulk-mode, shear-mode, or lateral-extension mode. In this work, we have used a lateral-extension-mode resonator that utilizes the d_31_ piezoelectric constant.

## 3. Design and Simulation

**Figure 3:**
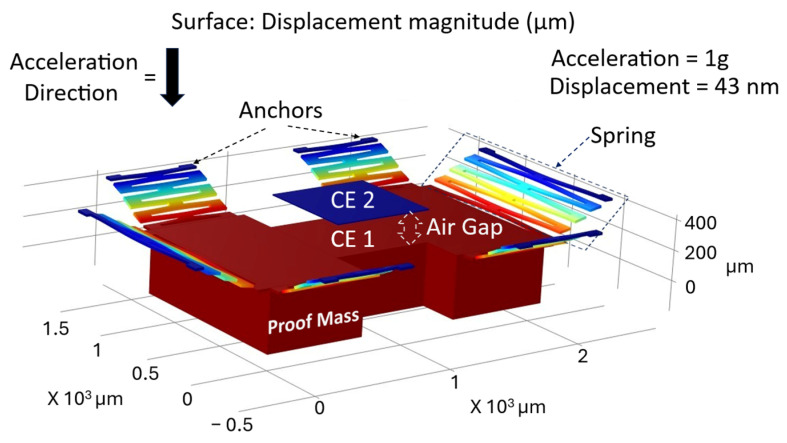
COMSOL image of a CMS structure showing the bottom capacitor electrode (CE 1) along with the proof mass, spring, and anchor, and the fixed top capacitor electrode (CE 2).

The sensor modeling and simulation are performed in a multi-step approach using finite element analysis (COMSOL 5.6 (AC/DC and Sructural Mechanics modules) software package). The first part of the simulation involved designing the CMS structure and calculating its displacement as a function of acceleration from 0.1 g to 100 g, and determining its natural resonance frequency, which determines the operational bandwidth. The displacement was simulated by applying acceleration to the CMS structure and sweeping the acceleration value. To substantially increase the mass associated with the sensor’s mass-spring structure and hence increase the sensitivity, the CMS structure is formed from both the device and the handle-layer silicon of a silicon-on-insulator (SOI) substrate (Figure 3). The springs are designed as folded doubly-clamped beams symmetrically arranged around the proof mass and formed from the top silicon device layer of the SOI substrate. The materials (properties) used for the simulation are Si (default) and AlScN (modified AlN properties) [37]. A fixed boundary condition is applied on the anchors for the simulations. The overall stiffness-to-mass ratio is tuned to facilitate a large range of input acceleration. For example, for a ~0.7 X ~0.7 mm2 CMS structure, 1 g of acceleration results in a ~43 nm displacement for the spring design depicted in Figure 3. The natural out-of-plane resonance frequency of the CMS structure was observed at ~2.391 kHz for the same structure. Given that the experimental testing frequency of our system (20 Hz) is far below the resonance frequency (2.391 kHz) of the CMS, the dynamic amplification factor is negligible.

**Figure 4:**
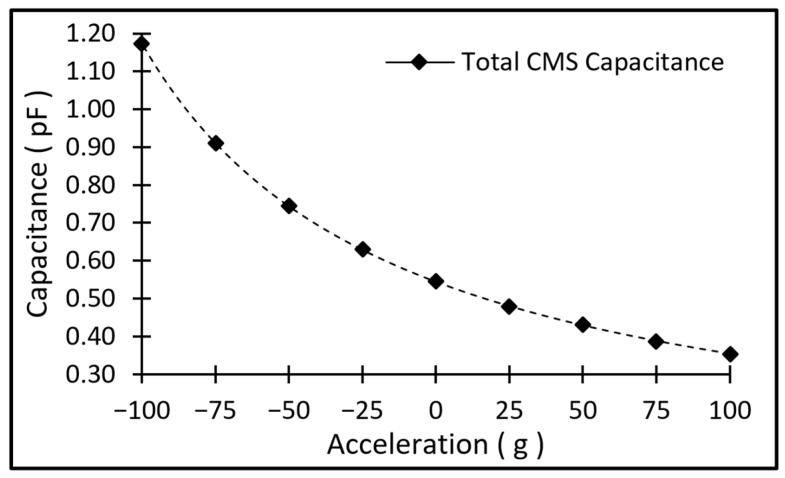
Change in capacitance value as a function of acceleration.

Using the displacement value and knowing the initial gap between the top and bottom electrodes of the CMS structure, the final gap can be calculated for a given input acceleration. Subsequently, the capacitance of the CMS structure can be derived as a function of input acceleration. This capacitance value is plotted for the nominal design shown in Figure 3, and can be seen in Figure 4. To evaluate the impact of fringing fields, a 3D electrostatic COMSOL model using air-box encapsulation was compared against the ideal parallel-plate theoretical model. For the nominal 7 µm gap, the FEA-calculated capacitance was 0.5612 pF, representing a 9.6% increase over the theoretical value of 0.5119 pF due to fringing effects. While the frequency-shift simulations utilized the theoretical capacitance model, this ~10% difference was relatively small, and the sensitivity depends on the change in capacitance rather than just the absolute base capacitance, so we opted to use the theoretical model, which provides an acceptable first-order approximation of the sensor’s performance.

**Figure 5:**
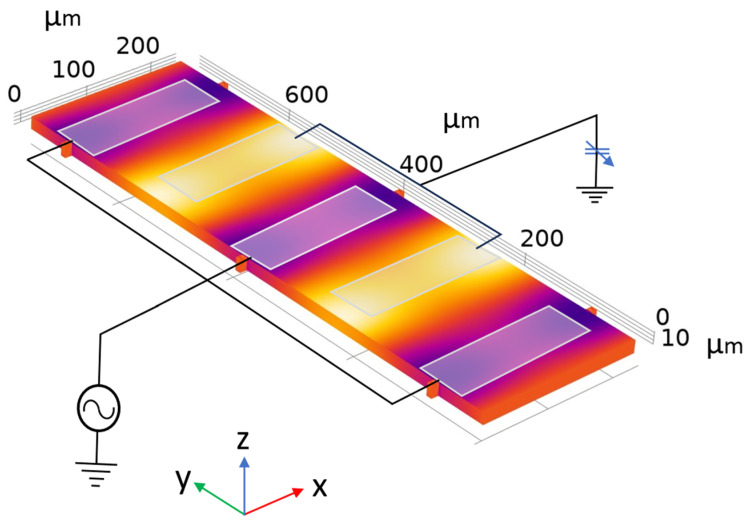
COMSOL image of a two-port resonator, resonating at ~26 MHz, with one port electrically connected to a variable capacitor and another electrically connected to a signal generator.

**Figure 6:**
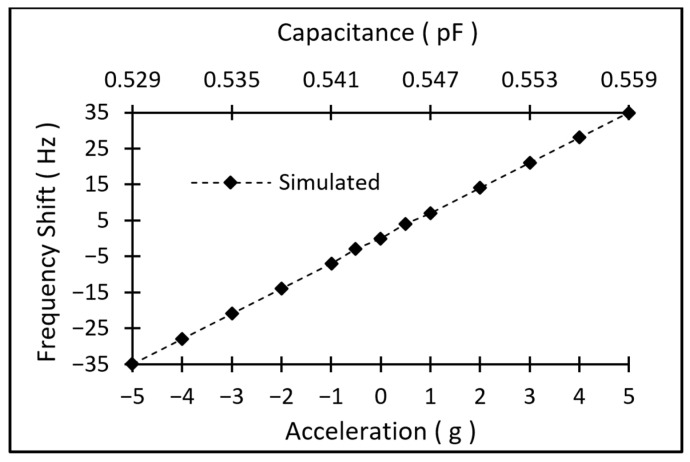
Simulated frequency shift of the accelerometer under ±5 g of acceleration, showing a ~7 Hz/g (~0.278 ppm/g) respectively.

The second part of the simulation involved the design of the resonator. A 5th-order thin-film piezoelectric-on-silicon (TPoS) resonator, with a 1 µm thick aluminum scandium nitride (AlScN) piezoelectric layer and 16 µm of silicon, operating at ~26 MHz, was designed (Figure 5). The device-layer thickness was chosen as 16 µm based on the wafer availability. After establishing the resonator dimensions for the target frequency, a lumped built-in capacitor element is added using the COMSOL AC/DC module, to terminate one port of the resonator. For this study, a frequency-domain analysis was performed in conjunction with a parametric sweep. COMSOL’s normal pre-defined mesh (min = 13.8 µm, max = 76.5 µm) was used, since the frequency difference between the normal and fine meshes was only 0.0054%. A shift in the resonator’s frequency was observed by varying the capacitance value (derived from the CMS simulation) in the parametric sweep section, as shown in Figure 6.

The resonance frequency is predicted to shift by ~14 Hz for a ±1 g acceleration, as shown in Figure 6 (~7 Hz/g sensitivity). Given that the displacement for the CMS structure is 43 nm/g, the sensor is suitable for high-precision acceleration measurement applications.

## 4. Fabrication Process

**Figure 7:**
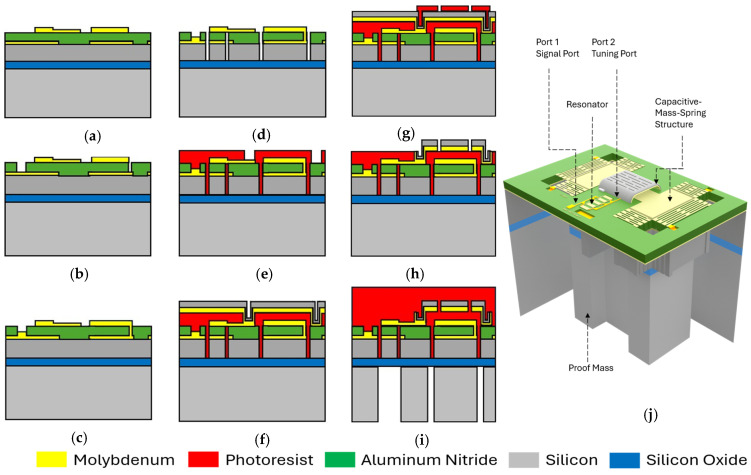
The schematic description of the sensor fabrication process flow. (**a**) The bottom Mo electrode deposition and patterning, followed by AlScN/Mo deposition and top electrode Mo patterning. (**b**) AlScN wet etching. (**c**) Mo, Cr, and Au stack deposition/patterning through lift-off. (**d**) Mo-AlScN-Mo-Si stack etch through sequential plasma etching followed by DRIE. (**e**) Sacrificial PR spinning and patterning. (**f**) Sputtering of the Ti-Mo-Si stack. (**g**) Patterning the Ti-Mo-Si stack to form the SFE. (**h**) Plasma etching of the Ti-Mo-Si stack. (**i**) DRIE of the handle layer to form the proof mass from the backside. (**j**) A 3D image of the accelerometer fully fabricated structure.

The entire fabrication process flow, schematically shown in Figure 7, was executed on a 4-inch silicon-on-insulator (SOI) wafer, consisting of a ~400 µm thick handle layer and a ~16 µm thick device layer. The process starts with sputtering ~100 nm of molybdenum (Mo) layer, which serves as the bottom electrode. The Mo layer was selectively etched in SF_6_ plasma to reduce the parasitic capacitance between the electrical line connecting the resonator’s second port to the suspended fixed electrode (SFE) of the CMS structures. The wafer was then sent to a commercial vendor to sputter a stack of ~1 µm AlScN and ~100 nm Mo.

Next, the top Mo layer was patterned to form the top electrodes and the bottom electrode of the CMS structure (Figure 7a). Subsequently, a wet etching was performed to access the underlying bottom metal through the AlScN layer (Figure 7b). This process involved alternating immersion of the wafer in two different solutions: a heated (80 °C) TMAH-based developer and sulfuric acid, until the underlying metal layer was exposed. Once the bottom metal was exposed, a new stack layer of ~250 nm Mo, ~20 nm chromium (Cr), and ~100 nm gold (Au) was selectively deposited by lift-off (Figure 7c). This stack is pivotal in establishing robust electrical connections between the bottom metal and the intricate SFE of the mass-spring structure.

Next, a plasma dry-etching process was performed using a combination of Cl_2_, BCl_3_, and Ar gases, to precisely etch through the Mo-AlScN-Mo stack followed by a deep reactive ion etching (DRIE) step to etch the device-layer silicon (Si), all using a silicon dioxide hard mask (Figure 7d). Next, a meticulously optimized lithography process followed, aiming at preparing the substrate for the consequent stack-layer deposition that would form the fixed electrode of the CMS, which also contacts the resonator’s top electrode through posts on the two sides. Given the challenging depth of the ~17 µm trenches on the substrate, the photoresist (PR) spinning process was finely tuned to ensure complete coverage while maintaining a uniform PR height of ~7 µm from the wafer surface. This optimization involved employing a burst spin technique with PR S1813 (Kayaku Advance Materials, Westborough, MA, USA), spun at 800 rpm for a brief duration of 5 s, resulting in a precisely controlled photoresist height of ~7 µm. The PR was then baked at 100 °C for 10 min to improve the adhesion and to withstand the long sputtering process (Figure 7e).

Next, a stack of titanium (Ti) (~40 nm), Mo (~750 nm), and Si (~750 nm) were sputtered onto the wafer surface (Figure 7f). Ti and Si were RF sputtered at 2 mtorr and 8.5 mtorr, respectively, at 100 W, while Mo was DC sputtered at 8.5 mtorr/50 W.

**Figure 8:**
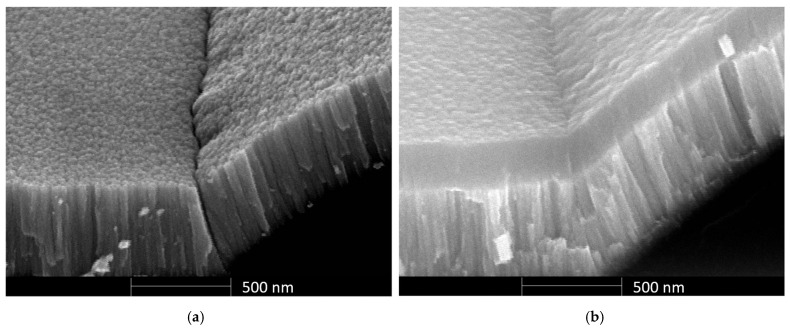
(**a**) A side-view image of the suspended fixed electrode (SFE) structure, showing the disconnect at the wafer–photoresist interface after the sputtering of molybdenum (Mo). (**b**) A similar side-view image of the suspended fixed electrode (SFE) structure, with no visible crack, after sputtering a stack of molybdenum (Mo) and silicon (Si).

The Mo layer in this stack is acting as the conductive electrode of the CMS structure. However, the polycrystalline grain boundaries in the Mo layer introduce high-stress points at the interface between the wafer surface and the photoresist, as can be seen in Figure 8a, promoting crack development and substantially compromising the process yield. To mitigate this, an amorphous Si layer was sputtered on top of the Mo, providing mechanical reinforcement, which also balances the stress on the Mo layer and enhances the overall structural integrity and durability, as can be seen in Figure 8b. A subsequent lithography, using AZ 3330F PR (Integrated Micro Materials, Argyle, TX, USA) with a spin-coated thickness of 4 µm, aimed at selectively etching away unwanted portions of the Ti-Si-Mo layer, was performed (Figure 7g), during which release holes were also defined in the intricate SFE structures (Figure 7h). The Ti-Mo-Si layer was dry-etched using SF_6_ and C_4_F_8_ gases at flow rates of 50 and 20 sccm, respectively, 15 mtorr chamber pressure, and 25 W power.

**Figure 9:**
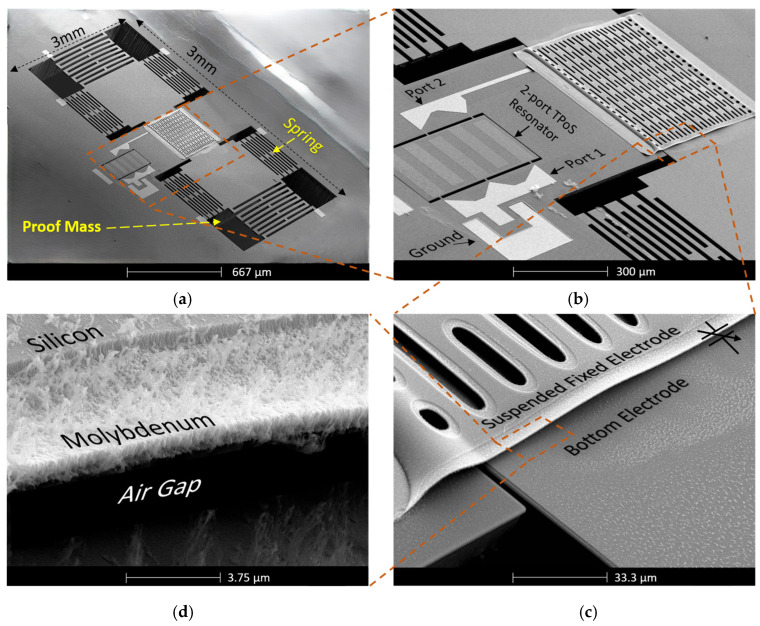
(**a**) A SEM image of a complete accelerometer. (**b**) A zoomed-in image of the accelerometer showing the CMS structure, electrical line, and the 2-port resonator. (**c**) A zoomed-in image of Figure (**b**), showing the top and the bottom electrodes. (**d**) Image of the composite SFE structure showing the Si and the Mo layer, and the air gap between the top and bottom electrodes.

The final lithography step targeted the wafer’s backside, enabling the release of the resonators and the formation of the proof mass (in the CMS structure) necessary to achieve a large mass-to-footprint ratio. DRIE was used to etch through the ~400 µm of the Si handle layer, fully releasing the resonators and forming the crucial proof mass (Figure 7i). A 12 µm thick AZ 10XT photoresist (Integrated Micro Materials, Argyle, TX, USA) served as the etch mask, and the process alternated SF_6_ (etch) and C_4_F_8_ (passivation) cycles. This resulted in an etch rate of 3.2 μm/min with an etch selectivity of more than 1:50 (PR: Si). Once completed, the wafer was broken into multiple dies. The final stage of the fabrication process involved etching the exposed BOX oxide layer and stripping the sacrificial PR layer supporting the SFE structures. The oxide layer was etched using buffered oxide etch (Figure 9d).

Finally, the sacrificial photoresist under the fixed capacitive electrode is etched in high-pressure (~700 mtorr), 65 W power O_2_ plasma to ensure the precise removal of the sacrificial PR layer without affecting the Si and the Mo layer (Figure 7j). The average etch time for the complete photoresist removal is ~4 h.

A detailed multi-part SEM image for an accelerometer is shown in Figure 9, highlighting the fully fabricated hybrid sensor architecture, the CMS structure, proof mass, 2-port TPoS resonator, the electrical connection between the CMS structure and the resonator, and the air gap between the top and the bottom electrodes.

## 5. Testing and Results

The testing procedure involved a multi-step process, starting with evaluating the resonators’ performance, followed by assembling an oscillator circuit on a PCB using the sensor’s resonator, which was then subjected to acceleration testing through rotation.

**Figure 10:**
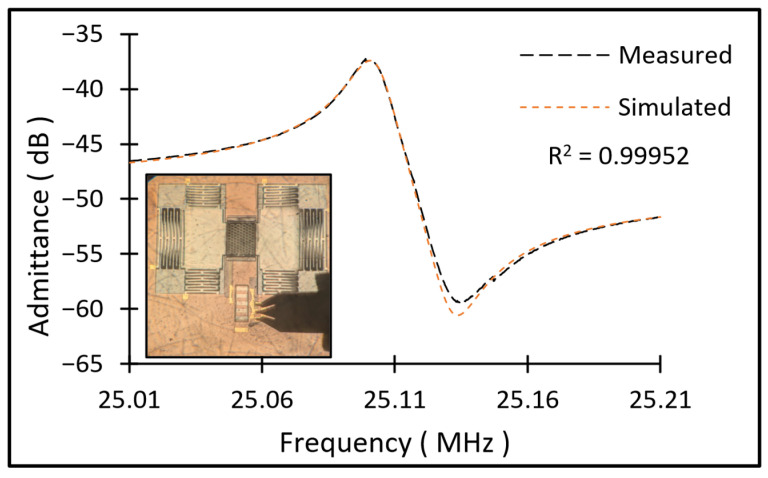
Measured and simulated admittance response (Y11), and the image of the resonator during its testing with a ground–signal–ground probe.

Frequency response (admittance) measured from a typical resonator using a vector network analyzer (VNA) is shown in Figure 10. The series resonance frequency of the resonator was recorded at ~25.1 MHz. The measured one-port S-parameters are utilized to develop the mBVD model of the resonator depicted in Figure 1b, and the derived lumped component values are listed in Table 1. The unloaded quality factor of the resonator is estimated as ~1548, and the coupling efficiency is ~0.27%. The simulated frequency response of the resonator using the mBVD model is overlaid on the measured results in Figure 10, proving a close match.

**Figure 11:**
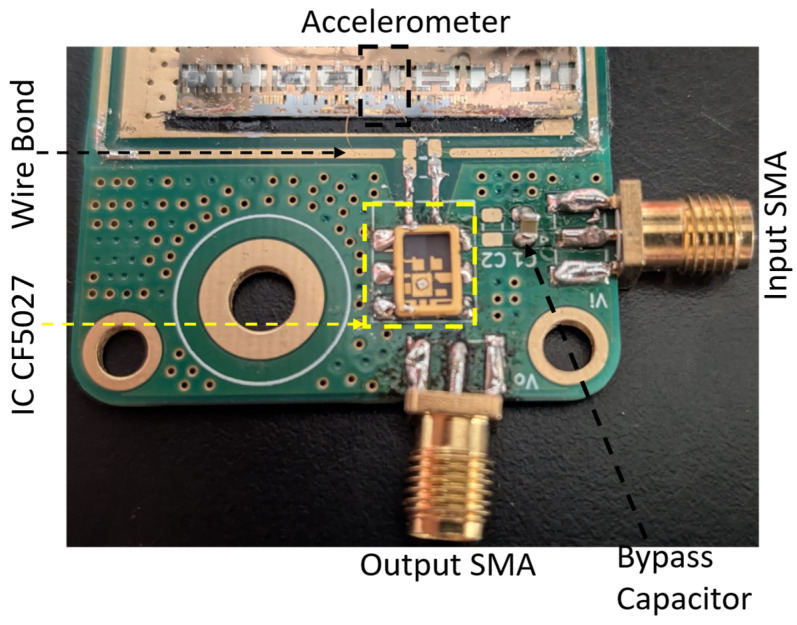
Image of the oscillator circuit containing the accelerometer electrically connected to IC CF5027 and its peripherals.

Once the resonator characterization was completed, the chip containing the sensor was mounted on a PCB designed for the oscillator assembly, and the resonator was wire bonded to the PCB, which contained a commercially available CF5027 IC to form an oscillator, as can be seen in Figure 11 [38]. Finally, it was verified that the SFE structure had survived the wire bonding process, before proceeding to the next steps.

**Figure 12:**
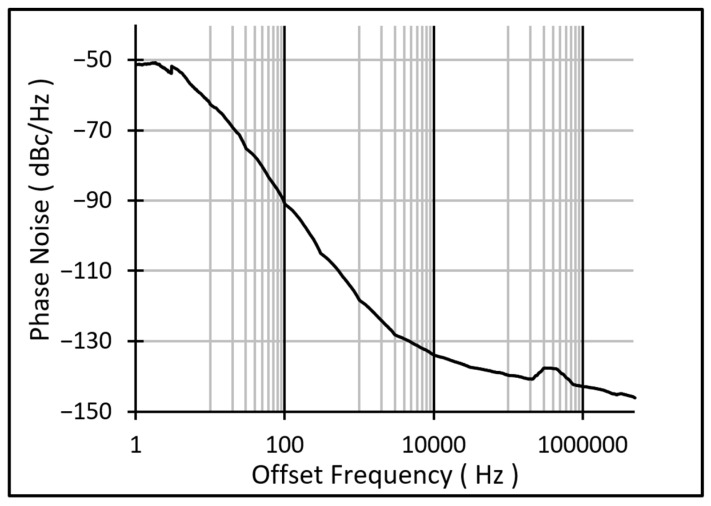
Phase noise analysis of the oscillator at 2.2 V.

**Figure 13:**
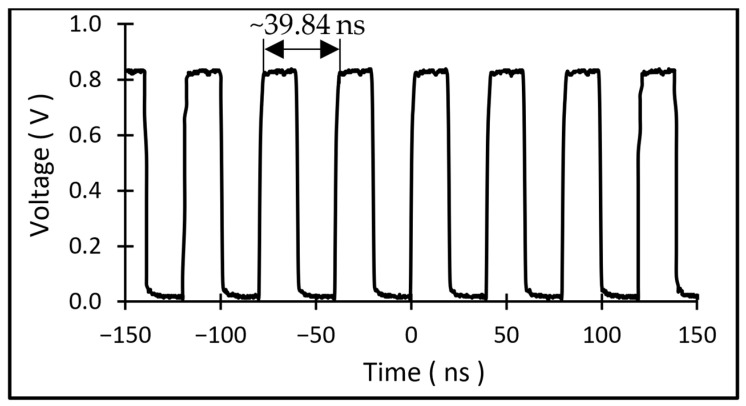
Oscillator waveform at 2.2 V input.

The phase noise of the resulting oscillator at a supply voltage of 2.2 V is shown in Figure 12, and the oscillator waveform is depicted in Figure 13. The oscillator exhibited a phase noise of ~−51.4 dBc/Hz at 1 Hz offset, which meets the requirement for measuring small frequency shifts. Based on the measured sensitivity of 6 Hz/g, this phase noise corresponds to a minimum detectable acceleration (MDA) of ~11.26 mg, calculated using (2) and (3) [38,39,40]:

(2) $$∆f\;=\;f_{0}\;(\sqrt{2(10^{\left(L\left(f\right)/10\right)})})$$

(3) $$MDA\;=\;\frac{∆f}{Sensitivity}$$

where ∆f is the frequency noise, $f_{0}$ is the resonance frequency, $L\left(f\right)$ is the phase noise, and MDA is the minimum detectable acceleration.

In this device, the phase noise is the limiting factor since the theoretical Brownian noise is calculated at 0.22 µg while the phase noise is 11,260 µg.

**Figure 14:**
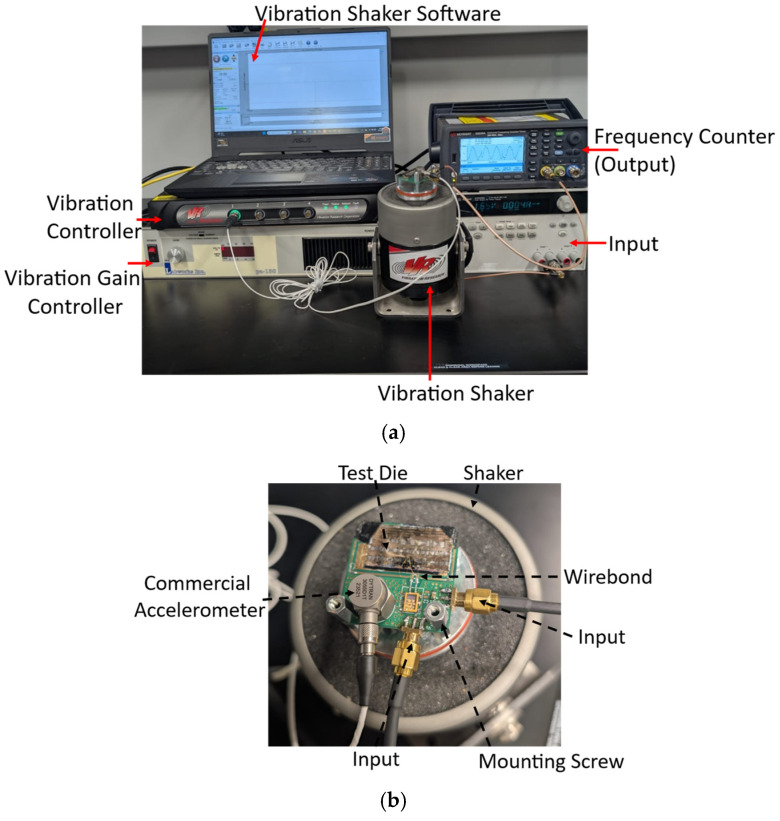
(**a**) Image of the accelerometer test setup using the shaker system. (**b**) Image of the accelerometer mounted onto the shaker.

**Figure 15:**
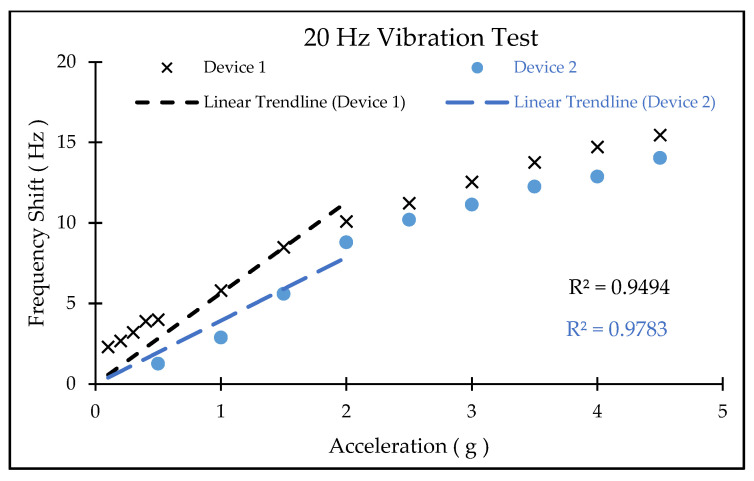
Measured frequency shift of two accelerometers under 0.1 g–4.5 g of acceleration. Devices 1 and 2 exhibited ~6 Hz/g (~0.23 ppm/g) and ~3.5 Hz/g (0.11 ppm/g) sensitivity, respectively. A linear trendline for devices 1 and 2, from 0 g to 2 g, shows sensitivities of 5.6429 Hz/g and 3.9347 Hz/g, respectively.

The oscillator was subsequently connected to a frequency counter for precise measurement of the sensor’s output frequency under 0.1 g to 4.5 g acceleration, applied using a shaker system (Figure 14) operating at 20 Hz in a room-temperature environment. Devices 1 and 2 exhibited a sensitivity of ~6 Hz/g (relative sensitivity of ~0.23 ppm/g) and ~3.5 Hz/g (relative sensitivity of ~0.11 ppm/g) (Figure 15), closely resembling the simulated prediction. The final sensitivity of the sensor is impacted by the final static gap between the SFE and the moving mass, which is hard to control. The presumed initial gap of 7 µm used in the simulation is an approximate thickness of the PR, but due to process non-uniformity and the residual stress, the actual gap is expected to be different from the presumption. The lower measured sensitivity of Device 2 is partially correlated to the smaller capacitive area and smaller overall mass.

A performance comparison with existing resonant accelerometer literature is summarized in Table 2. This work demonstrates a TPoS-based resonant accelerometer achieving a 5-fold increase in operational frequency (~25 MHz) over typical designs, while offering a clear pathway for scaling the operational frequency and sensitivity of the sensor.

## 6. Conclusions

This work introduces a novel design and fabrication paradigm for developing high-frequency accelerometers without compromising sensor sensitivity. An innovative process for fabricating large suspended structures for Z-axis sensing is demonstrated. Two functional accelerometers demonstrating high-frequency operation at ~25 MHz with sensitivities of ~6 Hz/g and ~3.5 Hz/g, respectively, were successfully realized and validated by finite element analysis and simulations, confirming a predicted sensitivity of ~7 Hz/g.

The hybrid design approach, which untangles the proof mass from the resonator, enables targeted design optimization for a range of accelerometer applications. For instance, the resonator frequency can be scaled without modifying the mass-spring structure, and vice versa.

The proposed design and the fabrication method can potentially result in the implementation of high-frequency, high-sensitivity MEMS accelerometers for passive wireless sensing, remote structural health monitoring, biomedical applications, and high-precision acceleration measurement applications in harsh environments.

## Figures and Tables

**Table 1:** mBVD model parameters.

mBVD Model	Value
R_s1_ (Ω)	0.2
R_m_ (Ω)	79.45
C_m_ (pF)	0.0517865
L_m_ (mH)	0.780057
C_0_ (pF)	0.011
C_p1_ (pF)	22.5
C_p2_ (pF)	10
C_CMS_ (pF)	0.544
R_s2_ (Ω)	1
kt^2^ (%)	0.27
Q (unloaded)	1548

**Table 2:** Sensor comparison.

Types of Accelerometers	Design	Operational Frequency	Sensitivity (Hz/g)	Resolution (mg)
Silicon Resonant Accelerometer [21]	Beam-shaped Differential	815 kHz	280	0.2
AlN-based Accelerometer [26]	DETF	890 kHz	3.4	12.73
Piezoelectric Accelerometer [41]	Flexural Vibrating Mode	1.1 MHz	73	7.5
BAW-based Accelerometer [28]	BAW	4.4 MHz	0.91	10
**This Work**	**Hybrid Design**	**25 MHz**	**6**	**100** **(~11 (Based on Equation (3)))**

## Data Availability

The data presented in this study are available on request from the corresponding author. The data are not publicly available due to privacy and on-going research.
